# Effect of Agricultural Beneficial Microbes on the Degradability of Polylactic Acid Film in the Farmland Environment

**DOI:** 10.3390/polym18020212

**Published:** 2026-01-13

**Authors:** Yuan He, Yi Dan, Long Jiang, Yun Huang, Hong Zhang, Yanjiao Qi

**Affiliations:** 1 State Key Laboratory of Advanced Polymer Materials, Polymer Research Institute, Sichuan University, Chengdu 610065, China; 18609495239@163.com (Y.H.);; 2 Gansu Province Research Center for Basic Sciences of Surface and Interface Chemistry, College of Chemical Engineering, Northwest Minzu University, Lanzhou 730124, China; 171941425@xbmu.edu.cn (H.Z.); 281101771@xbmu.edu.cn (Y.Q.)

**Keywords:** agricultural beneficial microbes, PLA film, degradation

## Abstract

Three common agricultural beneficial microbes, *Trichoderma harzianum*, *Bacillus cereus*, and *Pseudomonas fluorescens*, are widely used in the growth cycle of crops, and increase the yield of agricultural products through disease prevention and sterilization. As a biodegradable biological macromolecular material, polylactic acid (PLA) is also widely used in agricultural production as a biodegradable film. The addition of agricultural microbes will affect the degradation rate of polylactic acid and thus its agricultural use. Under specific conditions (Tri15), the degradation rate of PLA film exceeds 30%. Scanning electron microscopy (SEM) images show that the degradation of the PLA happened after 360 days of exposure to these three specific microbe environments, which makes the surface of PLA films crack. Gel permeation chromatography (GPC) analysis reveals that in the presence of these microbes, the molecular weight of PLA is reduced. The analysis of 16S rDNA sequences demonstrates that the introduction of these microbes alters the soil microbial community, resulting in an enhanced abundance of Betaproteomicrobes, promoting the degradation of PLA. These results indicate that the three microbes species significantly promote the degradation of PLA, and the effects of microbes vary for the different concentrations. This study establishes practical guidelines for the deployment of PLA in real-world farmland environments.

## 1. Introduction

Polylactic acid (PLA) is a biodegradable polyester synthesized primarily from lactic acid, which is derived from renewable plant resources, such as corn [1,2]. The starch from these plants undergoes saccharification to produce glucose, which is then fermented by specific strains of microorganisms to yield high-purity lactic acid, ultimately leading to the formation of PLA [3]. This material exhibits excellent mechanical properties and degradation characteristics, and capable of biodegradation, has been widely applied in various fields in recent years to address the environmental issues associated with traditional plastics [4,5].

Agricultural film is an essential agricultural practice designed to retain soil moisture and reduce water loss, leading to more efficient water resource utilization and enhanced crop productivity [6]. Traditional agricultural films are primarily composed of polyethylene (PE), a material characterized by a carbon-carbon single bond backbone that is hard to rapid degrade [7]. PE residues persist in soil, adversely affect soil properties and, subsequently, crop growth. Recently, efforts have been made to apply PLA in agricultural films to mitigate soil pollution issues resulting from the non-degradability of conventional polyethylene films [8]. As a biodegradable polymer, PLA will gradually degrade in soil environments, thus avoiding pollution. But if this degradation occurs too rapidly during the crop growth period, it could negatively impact crop yield by reducing moisture retention capabilities [9]. Therefore, when using biodegradable materials as agricultural films, it is crucial to consider their safe degradation period [10]. Currently, design and production of PLA biodegradable films have been considered to address this concern and practical applications reveal that PLA degradation is influenced by environmental factors [11]. Thus, the safe degradation period is affected not only by the product design but also the local environmental conditions. Researchers have examined the environmental influences on PLA degradation [12,13,14].

The findings from these environmental impact studies provide a solid theoretical foundation for the promotion and application of PLA films [15,16]. However, in practical agricultural experiments, it has been observed that studying temperature and humidity is insufficient. In the actual agricultural production, PLA degradation under identical environmental conditions varies, indicating that other factors may also affect PLA degradation [13,17,18]. Upon analyzing the agricultural growth environment, it was discovered that beneficial agricultural microorganisms, such as Bacillus subtitles and *Trichoderma harzianum*, are frequently employed to promote disease resistance and enhance root nutrition during crop growth [19,20,21]. These microorganisms are often directly sprayed onto the fields or used to coat seeds. As a biodegradable polymer, through microbial colonization, PLA degrades primarily on its surface, and then enzymes are secreted to facilitate the degradation process [22]. Thus, the presence of these beneficial microorganisms may alter the safety period of PLA films to influence the degradation of PLA [23].

This study primarily focuses on the effects of agricultural microorganisms on the degradation of PLA. And three common beneficial microorganisms are covered, including *Trichoderma harzianum*, *Bacillus cereus*, and *Pseudomonas fluorescens*. The scanning electron microscopy, Fourier transform infrared spectroscopy, and gel permeation chromatography are used to investigate the degradation impacts of these three microbes on PLA at the varied concentrations. Then, 16S rDNA gene sequencing is employed to assess the effects of these beneficial agricultural strains on the composition and diversity of soil microbial communities to reveal their potential mechanisms of action. This research aims to establish a solid theoretical basis to advance the subsequent application and promotion of PLA films in agriculture.

## 2. Materials and Methods

### 2.1. Materials

**Scheme 1:**
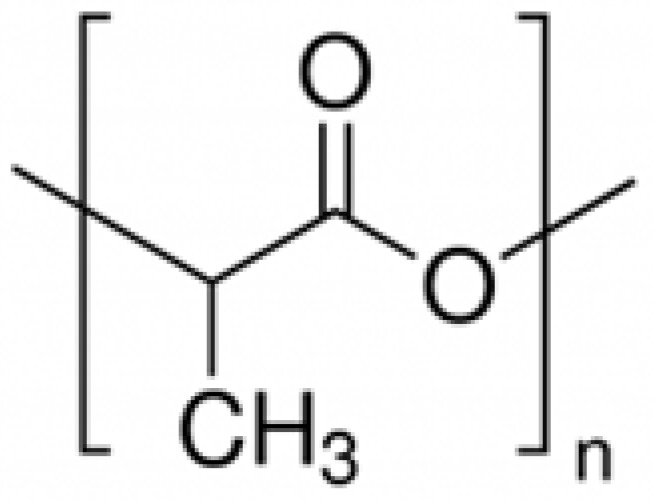
Chemical structure of PLA.

The polylactic acid (L106, ${\bar{M}}_{n}$: 6.81 × 10^4^, ${\bar{M}}_{w}$/${\bar{M}}_{n}$: 1.49) with the chemical structure shown in Scheme 1 was purchased from Wanhua Chemical Group Co., Ltd. (Yantai, China).

The soil used in this experiment was obtained from the topsoil of cultivated farmland in Binzhou, Shandong Province (37.22° N, 118.02° E). The initial properties of the soil were as follows: organic matter content, 7.43 g/kg, total nitrogen, 0.590 g/kg, moisture content, 32.7% and pH value of 6.8. It was classified as a sandy loam. The soil was dried at 80 °C in a vacuum oven for 8 h, followed by sieving through a 10-mesh (2 mm) sieve to exclude plant residues and coarse particles. This treatment effectively standardized the initial moisture while substantially reducing the native microbial background (Table S2) without significantly altering soil physicochemical properties.

This study employed *Bacillus cereus* (Bac), *Trichoderma harzianum* (Tri), and *Pseudomonas fluorescens* (Flu) as the beneficial microbes. The Bac and Tri strains originated from Bioworks, Inc.(Beijing, China)., and the Flu strain was acquired from Shandong Tainol Pharmaceutical Co., Ltd. (Weifang, China) Manufacturer specifications indicated that the products were mixtures of spores and vegetative cells, with viable counts of 8 × 10^8^, 3 × 10^8^, and 5 × 10^8^ CFU g^−1^ for Bac, Tri, and Flu, respectively. Based on drying at 105 °C until constant weight, the dry matter content of the powdered inoculants was determined to be 91% (Bac), 90% (Tri), and 93% (Flu). Prior to application, all microbial preparations were kept at 4 °C. The strains were used separately and were not subjected to co-cultivation.

### 2.2. Degradation Test of PLA Films with the Agricultural Beneficial Microbes

The PLA was vacuum-dried at 80 °C for 8 h to remove moisture. Films with a thickness of 500 ± 20 μm were then prepared by hot-pressing at 230 °C under 10 MPa. This thickness was selected to ensure sufficient mechanical integrity. The films were thus able to be handled and retrieved repeatedly without damage throughout the experiment. This durability allowed reliable sequential measurements, including mass loss and spectroscopic analyses, on the same samples.

A 500.0 ± 0.2 g portion of soil was weighed into each experimental vessel. Specified masses of each powdered inoculant (30 ± 0.1, 150 ± 0.1, 450 ± 0.1, or 900 ± 0.1 g) were first suspended in ultra-pure water and then manually homogenized into the soil. These masses corresponded to nominal concentrations of 1%, 5%, 15%, and 30% (*w*/*w*). Based on this concentration series, all samples were designated as follows: Tri1, Tri5, Tri15, Tri30; Bac1, Bac5, Bac15, Bac30; Flu1, Flu5, Flu15, Flu30. The prefix indicates the microbial strain (*Trichoderma*, *Bacillus cereus*, or *Pseudomonas fluorescens*), while the suffix indicates the nominal mass concentration. The corresponding absolute dosing values for viable cells (CFU g^−1^ dry soil) and microbial biomass (g dry weight g^−1^ dry soil) for each of these samples are provided in Table S1.

The soil burial experiment was carried out under controlled ambient laboratory conditions in Chengdu, Sichuan Province. No artificial temperature control was applied, allowing the indoor temperature to follow natural seasonal variations, the indoor recorded temperature from approximately 10 °C in winter (Jan) to 33 °C in summer (Jul–Aug). The soil vessels were exposed to ambient indoor lighting with a natural photo period but were protected from direct sunlight. Passive aeration was achieved through the vessel design, which included a loosely fitted lid and a maintained 10 cm head space above the soil. This configuration facilitated adequate gas exchange while minimizing moisture loss.

Prepared PLA film squares (0.5 mm thick, 4 cm side length) were buried at a consistent depth of 5 cm below the soil surface. Each container held four separate film pieces, arranged without contact or overlap. The control group received no microbial inoculates. All treatments were performed in triplicate.

**Scheme 2:**
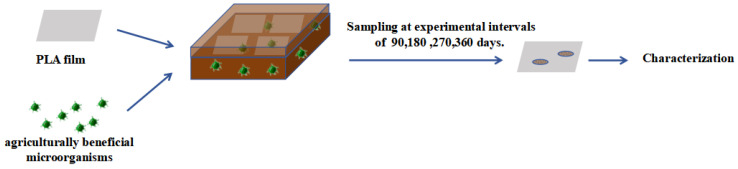
Degradation test of PLA films by the microbe.

The experiment lasted 360 days, with measurements taken every two weeks. Soil moisture was maintained at 30% (*w*/*w*) by monitoring levels with a handheld moisture meter and adding sterile ultra-pure water as needed during bi-weekly checks. PLA film samples were collected at 90, 180, 270, and 360 days. After retrieval, the films were gently cleaned with ethanol and prepared for analysis of surface morphology, average molecular weight, and other relevant properties. The overall experimental procedure is illustrated in Scheme 2.

### 2.3. Measurement of the Degradation Rate of PLA Film

First, the mass of PLA after degradation was determined following a specific processing protocol: the PLA films were separated from the soil, soaked in 70% ethanol for 30 min, washed three times with ultrapure water, and then dried to a constant weight at 60 °C.

Then, the degradation rate (D) was calculated by Equation (1):

(1) $$D=\frac{m_{0}-m_{t}}{m_{0}}\times 100\%$$

where m_0_ and m_t_ represent the mass of the initial PLA film and the PLA film after t days of degradation, respectively.

### 2.4. Structural Characterization of PLA Before and After Degradation

The morphology of the PLA films after various degradation periods was observed by scanning electron microscopy (SEM) with a Phenom G2 ProX scanning electron microscope (Eindhoven, North Brabant, The Netherlands) [24]. Before observation, the PLA films were coated with gold, and the test voltage was set at 10 kV.

The molecular structure was characterized by Fourier transform infrared (FTIR) spectroscopy using a Nichole iS10 instrument (Thermo Fisher Scientific Inc., Wilmington, DE, USA), covering the frequency range of 4000 to 400 cm^−1^ with a resolution of 4 cm^−1^ [25]. Fourier-transform infrared (FTIR) spectroscopy analysis was conducted using a spectrometer equipped with an ATR accessory. An automatic baseline correction was applied to all collected spectra.

The average molecular weight and molecular weight distribution of PLA were comprehensively analyzed by gel permeation chromatography (GPC, Waters, Milford, MA, USA), equipped with a Waters 1515 pump and a Waters 2414 refractive index detector. The PLA concentration was precisely maintained at 2 mg/mL, with tetrahydrofuran (THF) as the mobile phase at a flow rate of 1 mL/min. The measurements were performed at 35 °C, with narrow distribution polystyrene standards employed for system calibration.

### 2.5. Microbial Community Composition Analysis

The composition of the microbial community was analyzed by 16S_V3-V4 sequencing provided by Suzhou State and Biotechnology Co., Ltd. (Suzhou, China). [26].

## 3. Results

### 3.1. Effects of the Three Microbes on the Degradation Rate of PLA Films

**Figure 1:**
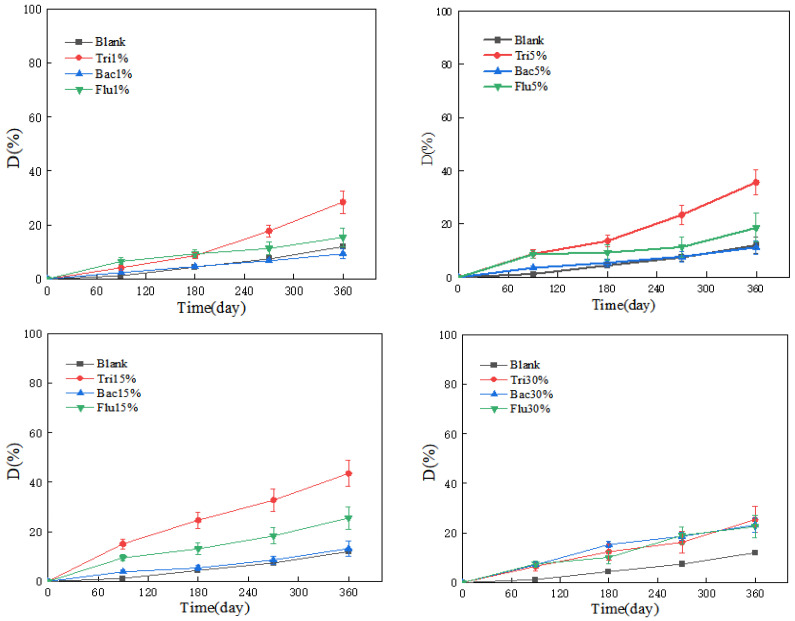
Variation in degradation rate (D) of PLA films as influenced by different microbes at different mass concentrations over time. Data points represent the mean ± SD (n = 3). Different lowercase letters at the same time point indicate statistically significant differences among treatments (*p* < 0.05).

The degradation rates of PLA films at different microbes concentrations were systematically investigated. The effect of the three microbes on the degradation of PLA films was evaluated according to the degradation rate of PLA films, and the results are presented in Figure 1 and Tables S1–S4.

Continuous degradation happens with all PLA films exhibiting some degree of mass loss over time. The degree of degradation promotion was strain-specific and concentration-dependent. On the whole, over time from 0 to 360 days, the degradation rate of PLA films under *Trichoderma harzianum* (Tri) was significantly increased. While the degradation rate growth with *Bacillus cereus* (Bac) is the weakest.

The three microbe strains exhibit varying degradation capabilities at different mass concentrations. Under the influence of Tri, as the concentration rises from 1% to 15%, the degradation rate of the PLA films was significantly increased. At a concentration of 15%, the degradation rate can achieve a maximum value of 42%. However, upon further increasing the concentration to 30%, the degradation rate diminishes by 20%. Under the presence of Flu and Bac, PLA membrane degradation rate increases with the rising concentration. At concentrations of 1% and 5%, Flu and Bac exhibited only a weak enhancing effect. When the concentration increases to 15% and 30%, both show a better promotion to PLA degradation, although the degradation promotion is still less effective than that under Tri.

Despite the varying effects of the three microbe strains on PLA degradation at different concentrations, it is evident that at a specific concentration level, all have accelerated the degradation rate of PLA during this time frame. This outcome clearly elucidates why the safety period for PLA is reduced under actual agricultural conditions. Different crops use different concentrations of strains, and therefore have different effects on PLA degradation. These effects may further affect its performance as a mulch film, thereby affecting crop growth [27]. So, in this study, the effects of three microbes on the structure and properties of polylactic acid membrane at different concentrations were further investigated.

### 3.2. The Change in Morphology of the PLA Films with Different Microbes

**Figure 2:**
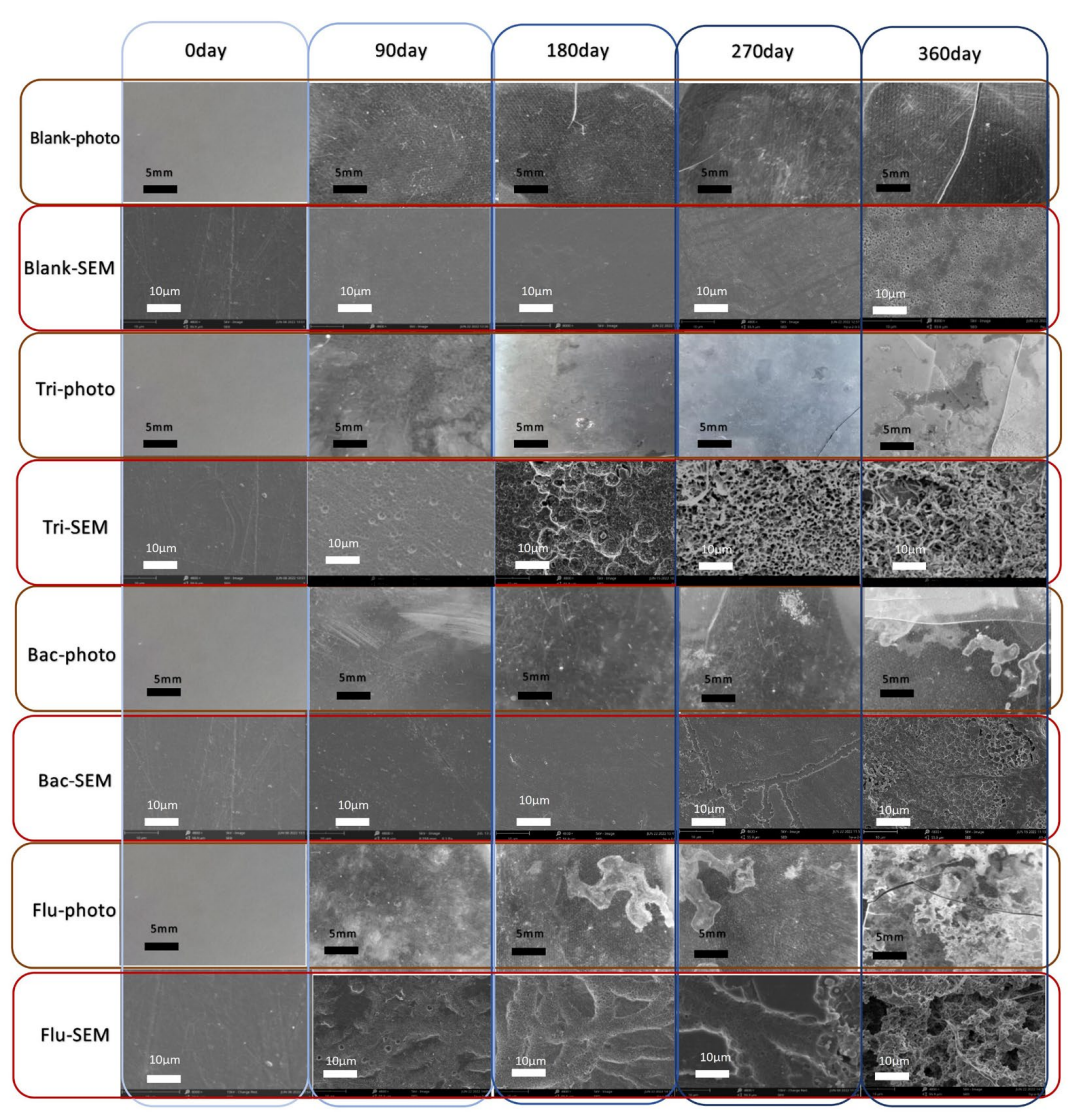
Macroscopic morphology photos (-photo) and scanning electron microscopy images (-SEM) of PLA films after the degradation test at different time points, under the influence of three different microbes with a concentration of 15% by mass, respectively.

The occurrence of degradation reduces the protective capacity of the membrane [28]. Figure 2 and Supporting Information Figures S1–S3 present macroscopic morphological photographs (Figure 2-Photo) and scanning electron microscopy images (Figure 2-SEM) of PLA film degradation in microbe environments. Before degradation, PLA films are transparent, accompanied by relatively flat, crack-free, and mildew-free surfaces shown by SEM images. At this time, PLA films have good protective properties. As the experiment progresses, small amounts of white spots and ruptures appear on the surface of the non-inoculated films at 360 days. And the three kinds of inoculated microbes samples undergo noticeable white discoloration and transparency decline with clear cracking and breakage, indicating that all three beneficial microbes significantly enhance the degradation of PLA, and the type and content of microbes exert varying degrees of damage to the micro structure of polylactic acid films.

Consistent with the degradation rate results, the degradation varies with the type of microbe strain and its content. *Trichoderma harzianum* has the best degradation effect in the PLA. The films underwent significant rupture and whitening at all concentrations. At a concentration of 15%, it exhibits the most substantial promotion to PLA degradation, resulting in the complete whitening of the PLA film and the breaks appear, which was observed in SEM. Only the growth of surface plaque can be observed at 30% concentration in SEM, indicating that the degradation promotion of this bacterium is not directly proportional to the concentration. *Pseudomonas fluorescens* also has the obvious effect of promoting the degradation of PLA. When the concentration is 1–30%, all colonies expand over time, eventually covering the entire film surface and leading to visible degradation. SEM observations reveal distinct fungal growth and extensive cracking. *Bacillus cereus* promotes a small amount of cracking on the PLA film surfaces and whitening at a concentration increase to 15% and 30%, the degradation of PLA films is relatively weaker at 1% and 5% of concentration. There was no significant change compared to the blank sample.

Despite the trend that the degradation of all PLA films increases with time, there are clear differences in the effect trends at the different concentrations by the three strains. At 90 and 270 days, most of the films under Bac did not change significantly except the film at 30% has a small amount of crack in SEM, which indicates that, from the perspective of agricultural production, the use of this beneficial bacterium has less impact on the application of PLA. For the other two microbes, some appearance of plaque and fine lines was observed starting at 90 days at four concentrations, and significant plaque and voids were observed at both the SEM and film surfaces at 180 days. When the time increases to more than 270 days, there is a more obvious degradation, including complete whitening of the film and severe rupture, which is evident at low concentrations of 1% and 5%, indicating that the use of these two beneficial microbes in agriculture will affect the use of PLA mulch.

Microscopically, the micro structure of the samples progressively deteriorates over time. SEM images reveal abundant pores, indicating surface-level degradation. The increase in pore quantity correlates with higher material loss rates and more advanced degradation [29]. Plaque growth over time is clearly observed in the samples, as evidenced in the Figures. These changes in surface morphology correspond with the previously observed degradation rate results. This also shows that the use of three kinds of microbes in agriculture will affect the safety period of poly lactic acid film, which we need to pay special attention to in real agricultural production.

### 3.3. Effect of Agricultural Beneficial Microbes on PLA Molecular Weight and Molecular Weight Distribution

Given that all three microbe strains effectively promote PLA degradation at a mass concentration of 15%, the average molecular weight and distribution of PLA under the action of the microbes at this concentration were analyzed by GPC. The results are presented in Table 1. Compared with the blank sample, the addition of the three strains decreased the molecular weight of PLA film and widened the molecular weight distribution. Different microbes have different promotion to the degradation of PLA. The decrease in molecular weight under the action of Tri was most obvious, and the molecular weight distribution was significantly widened, which was consistent with the previous value of degradation rate, indicating that 15% Tri had a better degradation effect on polylactic acid membrane, which is also consistent with the previous morphological observation results. Also, the data shows that as the degradation time increases, the average molecular weight of PLA decreases, and the molecular weight distribution becomes wider. These changes correspond with the results previously observed in degradation rates and morphological transformations.

### 3.4. Chemical Structure Analysis of PLA Films Under the Influence of Different Microbes

**Figure 3:**
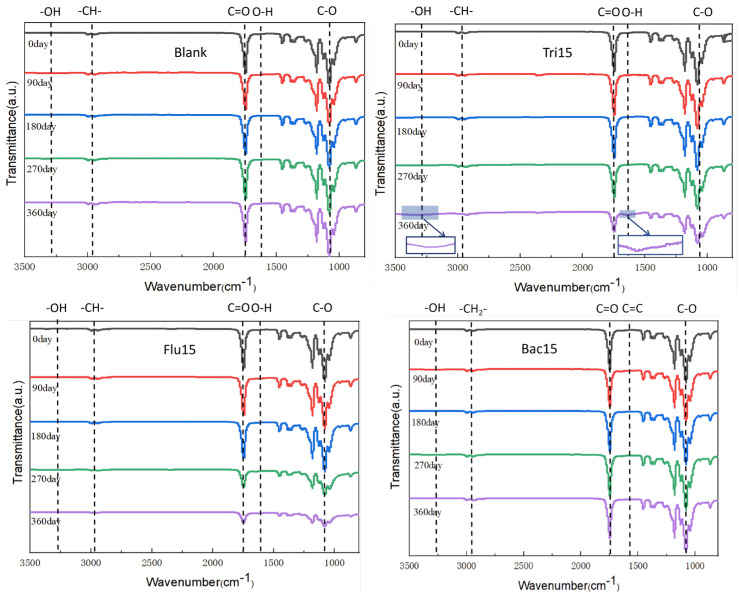
FTIR spectra of PLA films after degradation for different times under the action of different microbes (Blank: without microbes; Bac15: with microbes Bac of 15% mass concentration; Tri 15: with microbes Tri of 15% mass concentration; Flu 15: with microbes Flu of 15% mass concentration).

The structural changes in degraded PLA films were further investigated using Fourier transform infrared spectroscopy (FTIR). The results at a microbe concentration of 15% are shown in Figure 3, while results at microbe concentrations of 1%, 5%, and 30% are presented in Supporting Information in Figures S4–S6.

As depicted in Figure 3, the initial PLA film exhibits characteristic absorption peaks at 1748 cm^−1^ and 1078 cm^−1^. The peak at 1748 cm^−1^ corresponds to the stretching vibration of the ester carbonyl (C=O) groups, while the peak at 1078 cm^−1^ belongs to the C-O groups within the PLA structure. The structure of the blank samples remains relatively unchanged over time. The composition of the samples under Bac is not obvious, indicating less structural change in this state. Compared with the blank sample, as degradation progresses, the peaks of the Tri-treated and Flu-treated samples at 1748 cm^−1^ and 1078 cm^−1^ gradually diminish with the extension of burial time. After reaching up to 360 days, the result shows that the C-O and C=O bonds are being cleaved, suggesting that the Flu and Tri microbe strains significantly enhance the degradation of PLA at a 15% mass concentration. In the microbe treatment, the C-O and C=O peaks of the samples treated with Flu decrease dramatically, and these peaks almost disappear completely after 360 days. And the samples treated with Flu display the second-most substantial effect, whereas those treated with Bac show the least structural alteration. However, under further Tri treatment, the degradation was performed until 360 days, with new absorption peaks emerging at 3290 cm^−1^ and 1550 cm^−1^ corresponding to -OH and -COO^−^ functional groups by the hydrolysis of ester bonds, respectively. The formation of carboxylate and hydroxyl groups is attributed to the cleavage of ester bonds in the polylactic acid (PLA) polymer chain [30].

Figures S4–S6 reveals that the structural changes in the PLA vary with concentrations of the added microbe strains, the general trend aligns with the results of degradation rate and morphological characterization. For the samples under Bac-treated, there is no significant structural change at low concentrations, and a significant reduction in peaks at 1748 cm^−1^ and 1078 cm^−1^ occurs when the concentration reaches 30%. The PLA sample treated with Flu shows structural alterations with an increasing promotional effect as concentration rises, peaking at a concentration of 30%. The persistence of the peaks at 3290 cm^−1^ and 1550 cm^−1^ at the 30% concentration suggests that substantial ester bond scission occurred in the PLA, as evidenced by the formation of hydroxyl and carboxylate groups. In contrast, the PLA sample treated with Tri shows the most significant structural changes at a concentration of 15%.

Combining the aforementioned experimental results presented in this paper, the addition of all three microbe strains enhances the degradation of PLA, although the extent of this enhancement varies based on the microbe concentration. Flu exhibits a greater promoting effect as concentration increases, while Tri shows the most obvious degradation-promoting effect at a mass concentration of 15%. To further investigate the mechanism underlying these observations, it is essential to examine the influence of these microbe strains on the soil microbial community in greater detail.

### 3.5. Impact of Three Beneficial Microbes on Soil Microbial Community

**Figure 4:**
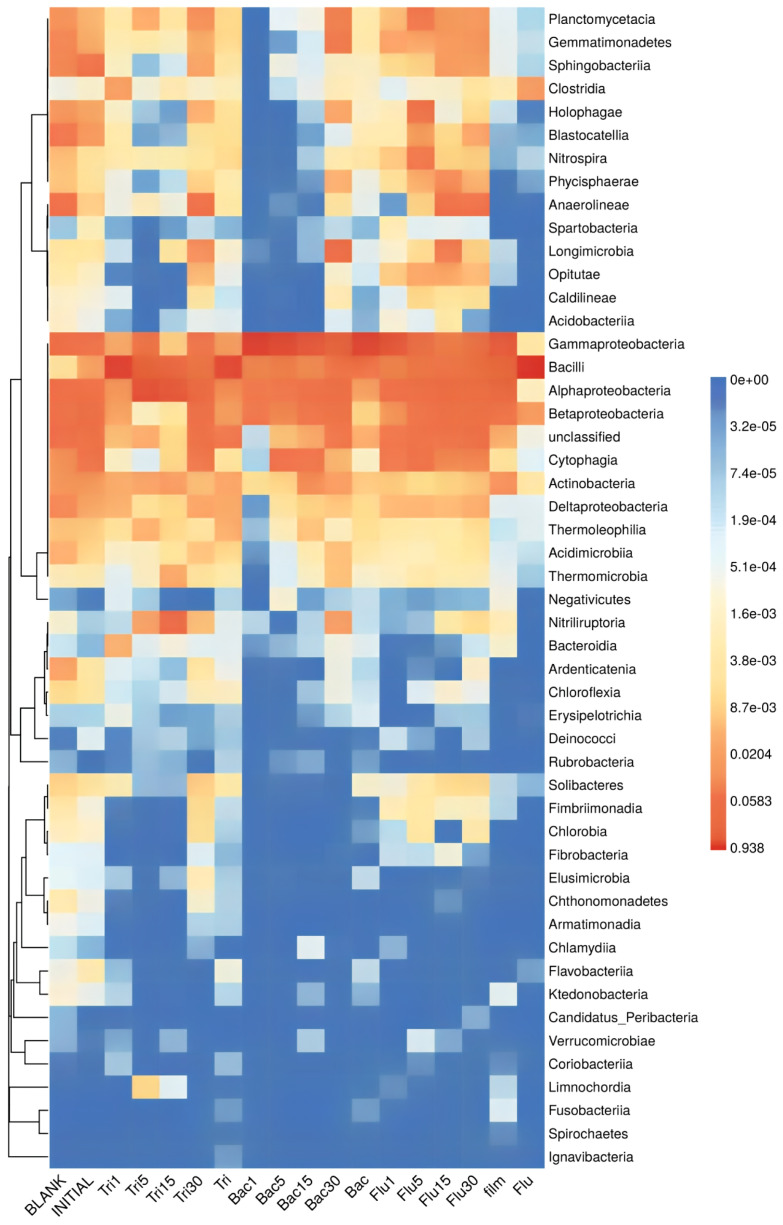
Soil microbes community composition and relative abundance at class level after PLA films degradation under the action of different microbes (Initial: soil before degradation; Blank: without microbes; Bac, Tri, and Flu are the Experimental microbes).

16S rRNA gene sequencing is utilized to evaluate the changes in soil microbiome after introducing the three different beneficial microbes, and the results of comparison of the microbial community composition are shown in Figure 4.

A comparison between the initial soil and the control soil containing suggested that the time had a limited influence on the soil microbial community. However, introducing the three beneficial microbial strains (*Bacillus cereus*, *Trichoderma harzianum*, and *Pseudomonas fluorescens*) appeared to lead to noticeable shifts in its composition. The addition of these microbes was associated with an increase in specific bacterial groups. In particular, the relative abundance of Alphaproteobacteria and Betaproteobacteria showed a marked rise. The concentration for achieving the greatest increase differed among the inoculants: the highest tested amount (30% *w*/*w*) of *B. cereus* and *P. fluorescens* was linked to the strongest effect, while for *T. harzianum*, a lower amount (15% *w*/*w*) appeared to be most effective. On the surface of the PLA film, Alphaproteobacteria and Betaproteobacteria were also identified as dominant groups, indicating they could play a significant role in the degradation process. The introduction of the beneficial microbes may have facilitated the growth of these groups in the soil, potentially supporting their colonization on the film. In summary, the observed enhancement in PLA degradation may not be directly caused by the added microbes themselves. Instead, it could be driven by their role in modifying the soil microbial community at specific concentrations, particularly by enriching Alphaproteobacteria and Betaproteobacteria, which might subsequently accelerate film breakdown.

## 4. Conclusions

Agricultural beneficial microbes, *Trichoderma harzianum*, *Bacillus cereus*, and *Pseudomonas fluorescens*, can change the soil microbial community and promote the degradation of PLA film under the soil environment. Therefore, we should pay attention to the impact of these microbes on the safety period when using these agriculturally beneficial microbes and PLA mulch simultaneously.

## Figures and Tables

**Table 1:** Average molecular weight and its distribution of PLA after degradation for different times under the influence of the three microbes at a concentration of 15% by mass.

Sample	Degradation Time (Day)	${\bar{M}}_{n}$ (×10^4^)	${\bar{M}}_{w}$ (×10^4^)	PDI
	0	6.81	10.13	1.49
Blank	90	5.17	7.93	1.53
	180	5.12	8.80	1.71
	270	4.93	8.31	1.68
	360	4.79	8.09	1.69
Tri15%	90	4.32	8.63	2.00
	180	4.38	7.14	1.63
	270	4.22	7.07	1.67
	360	4.13	7.01	1.70
Flu15%	90	5.81	9.01	1.55
	180	5.51	8.36	1.52
	270	5.48	8.52	1.55
	360	5.07	7.87	1.55
Bac15%	90	5.70	9.24	1.62
	180	5.68	8.00	1.41
	270	5.60	7.81	1.39
	360	5.47	7.76	1.42

## Data Availability

The original contributions presented in this study are included in the article/Supplementary Materials. Further inquiries can be directed to the corresponding author.

## Supplementary Materials

The following supporting information can be downloaded at: https://www.mdpi.com/article/10.3390/polym18020212/s1, Table S1: Absolute dosing of beneficial microbes in the soil degradation experiment; Table S2: Absolute dosing of beneficial microbes in the soil degradation experiment; Table S3: Statistical Analysis of PLA Degradation at 1% Mass Concentration; Table S4: Statistical Analysis of PBAT Degradation at 5% Mass Concentration; Table S5: Statistical Analysis of PBAT Degradation at 15% Mass Concentration; Table S6: Statistical Analysis of PBAT Degradation at 30% Mass Concentration; Figure S1: Macroscopic morphology photos (-photo) and scanning electron microscopy images (-SEM) of PLA films after the degradation test at different time points, under the influence of three different bacteria with a concentration of 1% by mass, respectively; Figure S2: Macroscopic morphology photos (-photo) and scanning electron microscopy images (-SEM) of PLA films after the degradation test at different time points, under the influence of three different bacteria with a concentration of 5% by mass, respectively; Figure S3: Macroscopic morphology photos (-photo) and scanning electron microscopy images (-SEM) of PLA films after the degradation test at different time points, under the influence of three different bacteria with a concentration of 30% by mass, respectively; Figure S4: FTIR spectra of PLA films after degradation for different times under the action of different bacteria (Bac 1%: with bacteria Bac of 1% mass concentration; Tri 1%: with bacteria Tri of 1% mass concentration; Flu 1%: with bacteria Flu of 1% mass concentration); Figure S5: FTIR spectra of PLA films after degradation for different times under the action of different bacteria (Bac5%: with bacteria Bac of 5% mass concentration; Tri 5%: with bacteria Tri of 5% mass concentration; Flu 5%: with bacteria Flu of 5% mass concentration); Figure S6: FTIR spectra of PLA films after degradation for different times under the action of different bacteria (Bac30%: with bacteria Bac of 30% mass concentration; Tri 30%: with bacteria Tri of 30% mass concentration; Flu 30%: with bacteria Flu of 30% mass concentration).

## Abbreviations

The following abbreviations are used in this manuscript:

PLA	polylactic acid
Tri	*Trichoderma harzianum*
Bac	*Bacillus cereus*
Flu	*Pseudomonas fluorescens*
SEM	Scanning electron microscopy
GPC	Gel permeation chromatography
FTIR	Fourier Transform Infrared Spectroscopy
D	the degradation rate
