# Supplementary material for: Effect of Agricultural Beneficial Microbes on the Degradability of Polylactic Acid Film in the Farmland Environment
Source: Polymers (Basel). 2026 Jan 13;18(2):212. doi: 10.3390/polym18020212 (PMC12846219; doi:10.3390/polym18020212)
Supplement: Supplementary file 1 [file polymers-18-00212-s001.zip › polymers-4066776-supplementary.pdf]

Supporting Information

Effect of agricultural beneficial microbes on the degradability of polylactic acid film in the farmland environment

Yuan He [1,2], Yi Dan,[1,*] Long Jiang[1],Yun Huanga[1], Hong Zhang[2], Yanjiao Qi[2]

[1] State Key Laboratory of Advanced Polymer Materials (Sichuan University), Polymer Research Institute of Sichuan University, Chengdu 610065, China; 18609495239@163.com

[2] Gansu Province Research Center for Basic Sciences of Surface and Interface Chemistry, College of Chemical Engineering, Northwest Minzu University, Lanzhou 730124, China

* Correspondence:Yi Dan, danyi@scu.edu.cn

Table S1. Absolute dosing of beneficial microbes in the soil degradation experiment

designate	m (g^{-1} dry soil)	CFU (g^{-1} dry soil)	g dry weight （g^{-1}dry soil）
Tri1	$(1\pm 0.01)\times10^{-2}$g	$(3\pm 0.03)\times10^{6}$	$(0.9\pm 0.01)\times10^{-2}$g
Tri5	$(5\pm 0.01)\times10^{-2}$g	$(1.5\pm 0.03)\times10^{7}$	$(4.5\pm 0.01)\times10^{-2}$g
Tri15	$(1.5\pm 0.01)\times10^{-1}$g	$(4.5\pm 0.03)\times10^{7}$	$(1.35\pm 0.01)\times10^{-1}$g
Tri30	$(3\pm 0.01)\times10^{-1}$g	$(9\pm 0.03)\times10^{7}$	$(2.7\pm 0.01)\times10^{-1}$g
Bac1	$(1\pm 0.01)\times10^{-2}$g	$(8\pm 0.05)\times10^{6}$	$(0.91\pm 0.01)\times10^{-2}$g
Bac5	$(5\pm 0.01)\times10^{-2}$g	$(4\pm 0.05)\times10^{7}$	$(4.55\pm 0.01)\times10^{-2}$g
Bac15	$(1.5\pm 0.01)\times10^{-1}$g	$(1.2\pm 0.05)\times10^{8}$	$(1.365\pm 0.01)\times10^{-1}$g
Bac30	$(3\pm 0.01)\times10^{-1}$g	$(2.4\pm 0.05)\times10^{8}$	$(2.73\pm 0.01)\times10^{-1}$g
Flu1	$(1\pm 0.01)\times10^{-2}$g	$(5\pm 0.05)\times10^{6}$	$(0.93\pm 0.01)\times10^{-2}$g
Flu5	$(5\pm 0.01)\times10^{-2}$g	$(2.5\pm 0.05)\times10^{7}$	$(4.65\pm 0.01)\times10^{-2}$g
Flu15	$(1.5\pm 0.01)\times10^{-1}$g	$(7.5\pm 0.05)\times10^{7}$	$(1.395\pm 0.01)\times10^{-}1$g
Flu30	$(3\pm 0.01)\times10^{-1}$g	$(1.5\pm 0.05)\times10^{8}$	$(2.79\pm 0.01)\times10^{-1}$g

Table S2. Absolute dosing of beneficial microbes in the soil degradation experiment

**sample**	native non-heated soil	sterilized soil	heat-treated + re-inoculated controls(Tri15)
**CFU (g^{-1} dry soil)**	$(7.10 \pm 0.01)\times 10^{8}$	$(2.70 \pm 0.01)\times 10^{3}$	$(4.50 \pm 0.03)\times 10^{7}$

Table S3. Statistical Analysis of PLA Degradation at 1% Mass Concentration

Time	sample	Mean degradation rates (D)%	SD (%)	p-value (Strain)	Tukey's HSD grouping
90 day	Blank	1.21	0.13	<0.05	c
	Tri1	4.22	0.82	<0.05	b
	Bac1	2.43	0.28	<0.05	bc
	Flu1	6.54	1.23	<0.05	a
180 day	Blank	4.45	0.82	<0.05	a
	Tri1	8.67	1.23	<0.05	b
	Bac1	4.65	0.42	<0.05	a
	Flu1	9.34	1.45	<0.05	b
270 day	Blank	7.43	1.27	<0.05	c
	Tri1	17.76	2.21	<0.05	a
	Bac1	6.68	0.58	<0.05	c
	Flu1	11.34	2.43	<0.05	b
360 day	Blank	12.03	3.13	<0.05	a
	Tri1	28.54	5.41	<0.05	b
	Bac1	9.34	1.83	<0.05	c
	Flu1	15.45	3.42	<0.05	ab

Table S4. Statistical Analysis of PBAT Degradation at 5% Mass Concentration

Time	sample	Mean degradation rates (D)%	SD (%)	p-value (Strain)	Tukey's HSD grouping
90 day	Blank	1.21	0.13	<0.05	a
	Tri5	8.78	1.79	<0.05	b
	Bac5	3.54	0.67	<0.05	a
	Flu5	8.67	1.45	<0.05	b
180 day	Blank	4.45	0.82	<0.05	a
	Tri5	13.65	2.01	<0.05	c
	Bac5	5.45	1.43	<0.05	a
	Flu5	9.35	2.87	<0.05	b
270 day	Blank	7.43	1.27	<0.05	a
	Tri5	23.45	3.56	<0.05	b
	Bac5	7.78	1.89	<0.05	a
	Flu5	11.34	2.78	<0.05	a
360 day	Blank	12.03	3.13	<0.05	a
	Tri5	35.64	4.78	<0.05	c
	Bac5	11.23	2.56	<0.05	a
	Flu5	18.56	5.42	<0.05	b

Table S5. Statistical Analysis of PBAT Degradation at 15% Mass Concentration

Time	sample	Mean degradation rates (D)%	SD (%)	p-value (Strain)	Tukey's HSD grouping
90 day	Blank	1.21	0.13	<0.05	a
	Tri15	15.13	2.02	<0.05	c
	Bac15	3.89	0.67	<0.05	a
	Flu15	9.54	1.23	<0.05	b
180 day	Blank	4.45	0.82	<0.05	a
	Tri15	24.67	3.23	<0.05	c
	Bac15	5.43	1.21	<0.05	a
	Flu15	13.15	2.45	<0.05	b
270 day	Blank	7.43	1.27	<0.05	a
	Tri15	32.74	4.51	<0.05	c
	Bac15	8.65	1.65	<0.05	a
	Flu15	18.36	3.34	<0.05	b
360 day	Blank	12.03	3.13	<0.05	a
	Tri15	43.56	5.24	<0.05	c
	Bac15	13.23	2.97	<0.05	a
	Flu15	25.57	4.42	<0.05	b

Table S6. Statistical Analysis of PBAT Degradation at 30% Mass Concentration

Time	sample	Mean degradation rates (D)%	SD (%)	p-value (Strain)	Tukey's HSD grouping
90 day	Blank	1.23	0.13	<0.05	a
	Tri30	6.55	2.02	<0.05	b
	Bac30	7.19	0.97	<0.05	b
	Flu30	7.54	1.23	<0.05	b
180 day	Blank	4.45	0.82	<0.05	a
	Tri30	12.45	3.23	<0.05	b
	Bac30	15.35	1.21	<0.05	c
	Flu30	10.15	2.45	<0.05	b
270 day	Blank	7.43	1.27	<0.05	a
	Tri30	16.28	4.51	<0.05	b
	Bac30	18.65	1.65	<0.05	b
	Flu30	19.04	3.34	<0.05	b
360 day	Blank	12.03	3.13	<0.05	a
	Tri30	25.34	5.24	<0.05	b
	Bac30	23.23	2.97	<0.05	b
	Flu30	22.57	4.42	<0.05	b

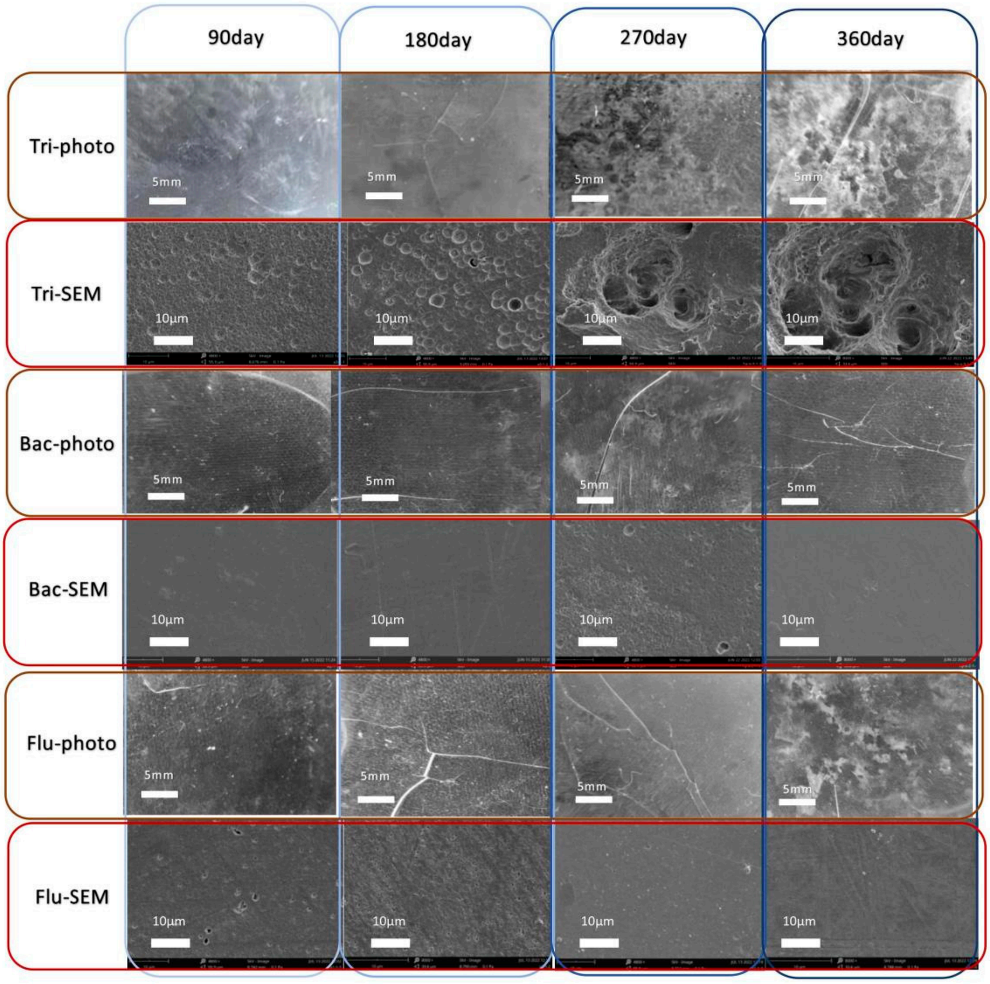

Figure S1. Macroscopic morphology photos (-photo) and scanning electron microscopy images (-SEM) of PLA films after the degradation test at different time points, under the influence of three different microbes with a concentration of 1% by mass, respectively.

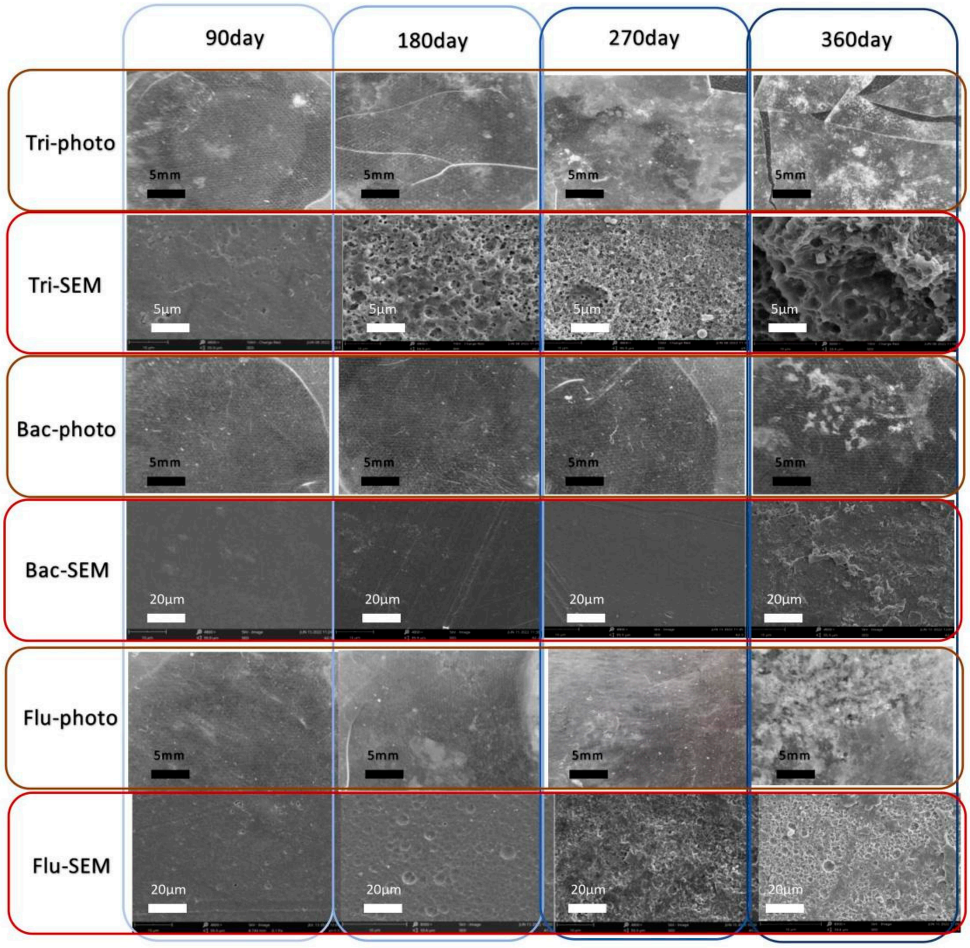

Figure S2. Macroscopic morphology photos (-photo) and scanning electron microscopy images (-SEM) of PLA films after the degradation test at different time points, under the influence of three different microbes with a concentration of 5% by mass, respectively.

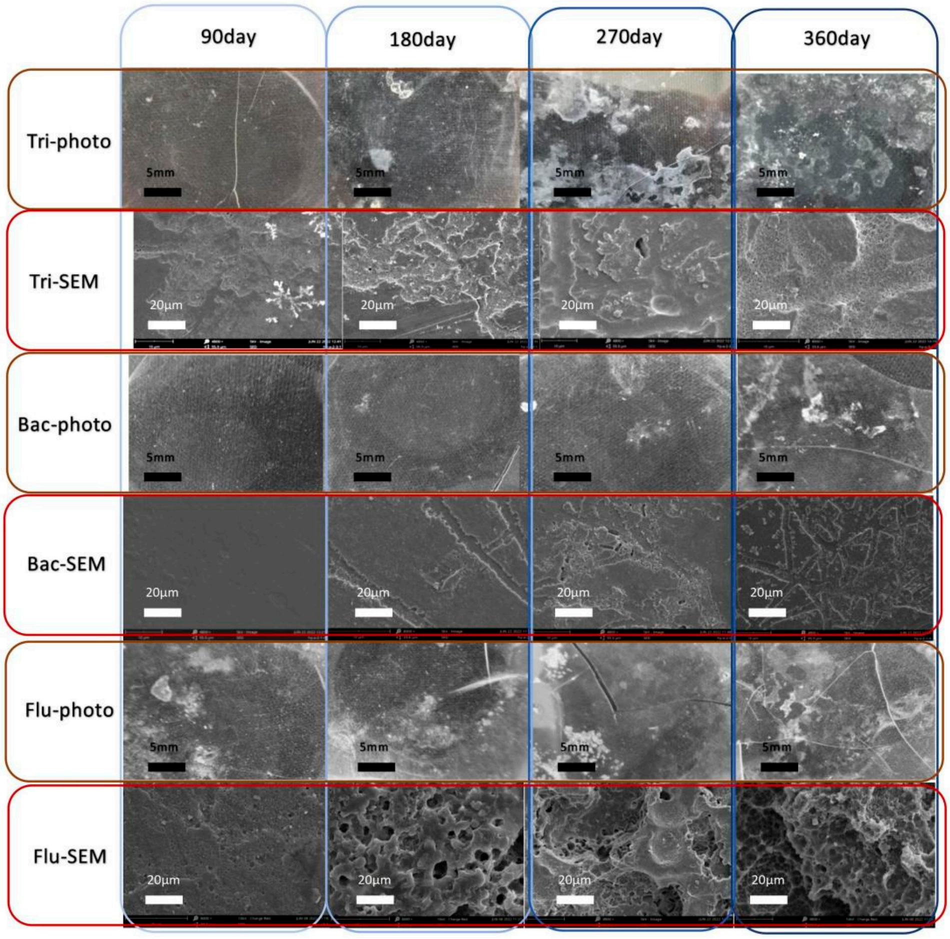

Figure S3. Macroscopic morphology photos (-photo) and scanning electron microscopy images (-SEM) of PLA films after the degradation test at different time points, under the influence of three different microbes with a concentration of 30% by mass, respectively.

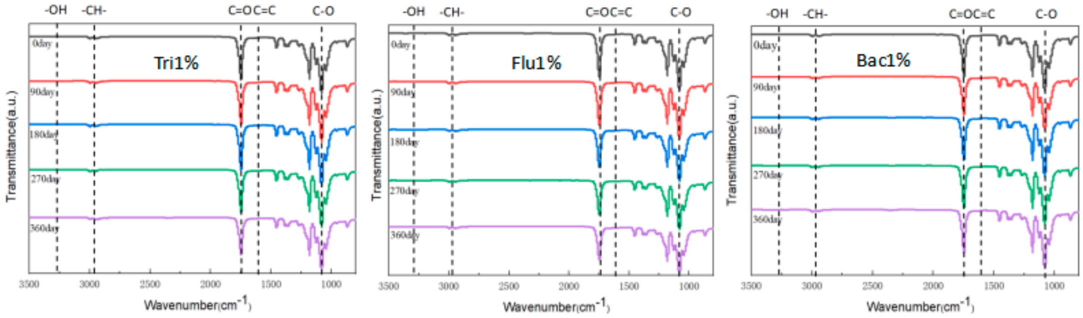

Figure S4. FTIR spectra of PLA films after degradation for different times under the action of different microbes (Bac1%: with microbes Bac of 1% mass concentration; Tri 1%: with microbes Tri of 1% mass concentration; Flu 1%: with microbes Flu of 1% mass concentration)

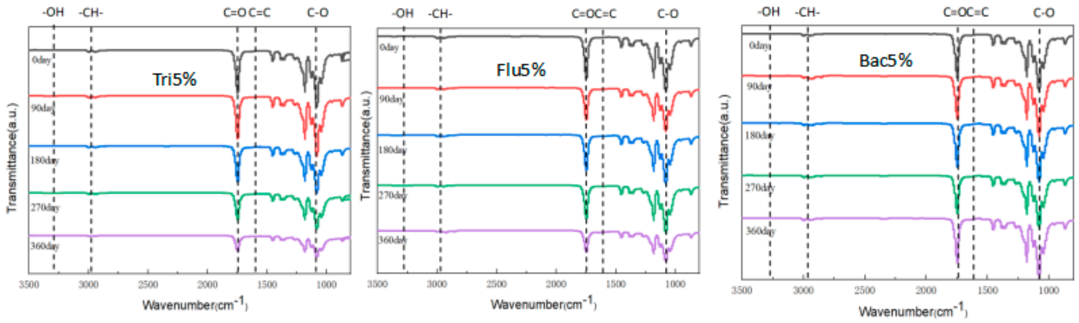

Figure S5. FTIR spectra of PLA films after degradation for different times under the action of different microbes (Bac5%: with microbes Bac of 5% mass concentration; Tri 5%: with microbes Tri of 5% mass concentration; Flu 5%: with microbes Flu of 5% mass concentration)

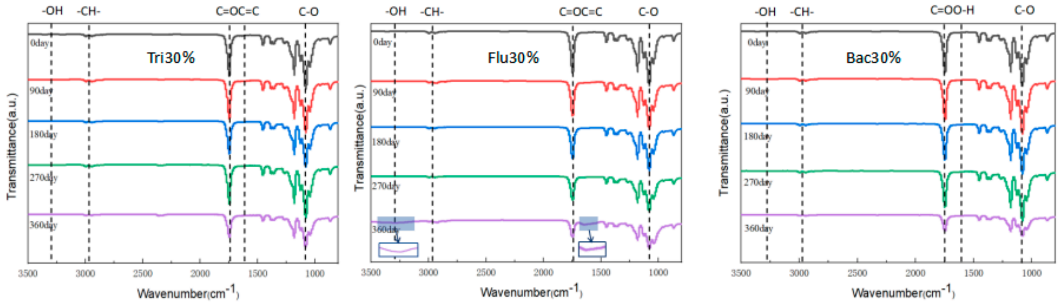

Figure S6. FTIR spectra of PLA films after degradation for different times under the action of different microbes (Bac30%: with microbes Bac of 30% mass concentration; Tri 30%: with microbes Tri of 30% mass concentration; Flu 30%: with microbes Flu of 30% mass concentration)
